# Enhancing mHealth Technology in the Patient-Centered Medical Home Environment to Activate Patients With Type 2 Diabetes: A Multisite Feasibility Study Protocol

**DOI:** 10.2196/resprot.6993

**Published:** 2017-03-06

**Authors:** Ronald Gimbel, Lu Shi, Joel E Williams, Cheryl J Dye, Liwei Chen, Paul Crawford, Eric A Shry, Sarah F Griffin, Karyn O Jones, Windsor W Sherrill, Khoa Truong, Jeanette R Little, Karen W Edwards, Marie Hing, Jennie B Moss

**Affiliations:** ^1^ Department of Public Health Sciences Clemson University Clemson, SC United States; ^2^ Nellis Family Medicine Residency Program Mike O'Callaghan Federal Hospital Las Vegas, NV United States; ^3^ Madigan Army Medical Center Tacoma, WA United States; ^4^ MHIC Laboratory Lead Telemedicine & Advanced Technology Research Center U.S. Army Medical Research & Materials Command Fort Gordon, GA United States

**Keywords:** mHealth, diabetes mellitus, patient activation, patient-centered medical home, patient centered care, eHealth, health information

## Abstract

**Background:**

The potential of mHealth technologies in the care of patients with diabetes and other chronic conditions has captured the attention of clinicians and researchers. Efforts to date have incorporated a variety of tools and techniques, including Web-based portals, short message service (SMS) text messaging, remote collection of biometric data, electronic coaching, electronic-based health education, secure email communication between visits, and electronic collection of lifestyle and quality-of-life surveys. Each of these tools, used alone or in combination, have demonstrated varying degrees of effectiveness. Some of the more promising results have been demonstrated using regular collection of biometric devices, SMS text messaging, secure email communication with clinical teams, and regular reporting of quality-of-life variables. In this study, we seek to incorporate several of the most promising mHealth capabilities in a patient-centered medical home (PCMH) workflow.

**Objective:**

We aim to address underlying technology needs and gaps related to the use of mHealth technology and the activation of patients living with type 2 diabetes. Stated differently, we enable supporting technologies while seeking to influence patient activation and self-care activities.

**Methods:**

This is a multisite phased study, conducted within the US Military Health System, that includes a user-centered design phase and a PCMH-based feasibility trial. In phase 1, we will assess both patient and provider preferences regarding the enhancement of the enabling technology capabilities for type 2 diabetes chronic care management. Phase 2 research will be a single-blinded 12-month feasibility study that incorporates randomization principles. Phase 2 research will seek to improve patient activation and self-care activities through the use of the Mobile Health Care Environment with tailored behavioral messaging. The primary outcome measure is the Patient Activation Measure scores. Secondary outcome measures are Summary of Diabetes Self-care Activities Measure scores, clinical measures, comorbid conditions, health services resource consumption, and technology system usage statistics.

**Results:**

We have completed phase 1 data collection. Formal analysis of phase 1 data has not been completed. We have obtained institutional review board approval and began phase 1 research in late fall 2016.

**Conclusions:**

The study hypotheses suggest that patients can, and will, improve their activation in chronic care management. Improved activation should translate into improved diabetes self-care. Expected benefits of this research to the scientific community and health care services include improved understanding of how to leverage mHealth technology to activate patients living with type 2 diabetes in self-management behaviors. The research will shed light on implementation strategies in integrating mHealth into the clinical workflow of the PCMH setting.

**Trial Registration:**

ClinicalTrials.gov NCT02949037. https://clinicaltrials.gov/ct2/show/NCT02949037. (Archived by WebCite at http://www.webcitation.org/6oRyDzqei)

## Introduction

Diabetes mellitus is a chronic disease with high rates of disability, impaired quality of life, and premature death [1-4]. The prevalence of type 2 diabetes is increasing at an alarming rate in the United States; in 2013, the estimated number of patients was between 20 million and 27 million, or about 7% to 10% of the adult population [2,3]. Research suggests that, if current trends continue, diabetes will be diagnosed in 1 in 3 adults in the United States by 2050 [4,5]. Diabetes is the leading cause of blindness, nontraumatic amputations, and adult renal failure, and reduces life expectancy by 5-10 years [2]. The individual symptom burden (eg, chronic pain, neuropathy, depression, and physical disability) is substantial and significantly increases in the older adult population [1]. In the United States, an average individual with diabetes incurs medical expenditures of about US $13,700 a year, of which about US $7900 is attributable to diabetes [4]. This represents an expenditure about 2.3 times greater than that for a diabetes-free individual [4].

Numerous primary care-based efforts have been aimed at reducing both the disease burden on individuals and the cost of diabetes care. A contemporary strategy is the management of patients with diabetes within the context of the patient-centered medical home (PCMH) setting. A key PCMH principle is the appropriate use of information technology to support optimal patient care, performance measurement, patient education, and enhanced communication [6]. Several case studies from various US health systems show the benefit of the PCMH model to improved diabetes care [7]. There is published evidence on the positive impact of PCMH-based care in psychosocial outcomes of patients with diabetes [8].

The potential of mHealth technologies in the care of patients with diabetes and other chronic conditions has captured the attention of clinicians and researchers. Efforts to date have incorporated a variety of tools and techniques, including Web-based portals [9-11], short message service (SMS) text messaging [9,12-14], remote collection of biometric data [12,15], electronic coaching [14], electronic-based health education [13], secure email communication between visits [16-18], electronic collection of lifestyle and quality-of-life surveys, and personal health records (PHRs). Each of these tools, used alone or in combination, has demonstrated varying degrees of effectiveness. Some of the more promising results have been demonstrated using regular collection of biometric devices (eg, glucometers, activity monitors) [12], SMS text messaging [12-14], secure email communication with clinicians and clinical teams [9,16,17], and regular reporting of quality-of-life variables aligned with decision support. In this study, we seek to incorporate many of the most promising mHealth capabilities in a PCMH workflow led by a clinical advisory team. We aim to address underlying technology needs and gaps related to the use of mHealth technologies and the activation of patients with type 2 diabetes.

### The Concept of Patient Activation

Self-management for patients with type 2 diabetes and other chronic conditions includes following complex treatment regimens, monitoring chronic conditions, and making lifestyle changes [19-22]. The chronic care model suggests that activated patients are better able to function in the role of self-manager [21,23]. An activated patient has the motivation, confidence, and skills necessary to enact behavioral changes and make health-related decisions [24-27]. These patients ask questions and collaborate with their health care provider [19,26-28]. Research shows that activated patients have more positive clinical outcomes, are more likely to receive preventive care, and have lower health care-related costs [24,26,29].

A recommended strategy in patient activation is the concept of “preactivating” patients prior to clinical encounters [20,30]. The concept incorporates active targeted communication and follow-up from the health care team [30]. Interventions to include educational programs [31], care coaching [32], and motivational interviewing [33] have been attempted to improve patient activation with varied success [34]. However, these efforts have infrequently been tailored to potential intrinsic differences in how the patients approach their disease. Theoretically, research suggests that patient activation can be increased [19,35-37]. Conceptualizing activation as a dynamic variable allows researchers to target this motivating factor that can potentially influence health behaviors [21,24,38,39].

### Previous Research on Patient Portals, Personal Health Records, Patient Activation, and Improved Outcomes

Federal legislation and movement toward patient centeredness in the United States has fueled interest in providing patients with access to their health information, enhanced communication with clinical environments, and greater emphasis on self-care [40-43]. Early research on portal and PHR use and patient activation provided mixed results. Several studies reported a positive significant relationship between use of portals and PHRs and activation of patients [41,43-45], while other studies did not realize a significant finding [40,46,47]. The design of these published studies prevented any in-depth inquiry into why (or not) portal and PHR use influenced patient activation. Their authors posited a variety of possible factors, including the target patient population [44], time since severe diagnosis or symptoms and episodes [46,47], and patient age (activation being higher in adults than in children) [45]. One study suggested that tailoring a portal or PHR intervention to the patient activation level may optimize intervention efficiency [43].

Early research on increased activation and improved clinical outcomes using patient portal and PHR-based interventions has also provided mixed results. Several studies demonstrated a relationship between increased patient activation and improved intermediate clinical outcomes (eg, hypertension, smoking, body mass index, and glycated hemoglobin [HbA_1c_]) [48], while a major study did not record a significant finding regarding the same outcomes [42]. It is noteworthy that these early studies did not provide substantial detail on design issues related to the portal or PHR, or whether the intervention included behavioral reinforcement.

### User-Centered Design

Design science will inform our development and testing [49,50]. User-centered design will guide development, following participatory design methods to understand more specifically how patients experience diabetes on a daily basis, what clinicians need to know from patients, and how to create a shared communication system for better decision making [51]. Consistent with the guidelines set forth by the Science Panel on Interactive Communication and Health [52], our evaluation design will incorporate the 3 elements of formative, process, and outcome evaluation. Methods include (1) clinician focus groups and in-depth patient interviews to define key knowledge variables that are personally and clinically relevant, (2) iterative usability testing with patients, and (3) iterative observations of the system in clinical settings [53].

### Military Health System: An Overview

The US Military Health System (MHS) is a large integrated health system that cares for about 9.39 million beneficiaries through its TRICARE insurance product and its substantial direct care system consisting of tertiary facilities, community hospitals, and clinics globally. Nearly 35% of its beneficiary pool are active duty members and their dependents, with a larger population (about 56%) being retirees and their beneficiaries [54]. The MHS direct care system is robust. Facilities are accredited by the Joint Commission (formerly the Joint Commission on Accreditation of Healthcare Organizations), and the MHS operates a dedicated educational infrastructure to support medical and nursing education programs [54]. The MHS has a connecting health information technology infrastructure to support clinical care and clinical operations.

### Hypotheses

Hypothesis 1: User-centered design will allow developers to create a patient-centered interactive and tailored mobile technology for use in the PCMH setting.

Hypothesis 2: The use of interactive and tailored mobile technology, the Mobile Health Care Environment (MHCE), employed in the PCMH setting will increase the activation of patients with chronic type 2 diabetes.

Hypothesis 3: The use of interactive and tailored mobile technology in a PCMH setting will increase diabetes self-care activities.

Hypothesis 4: Patients who engage at a higher rate with the interactive and tailored mobile technology in a PCMH setting will realize greater improvement in clinical measures.

The primary goal of the research is to enhance patient activation levels and improve self-management of type 2 diabetes through the use of the MHCE in the PCMH setting. While there are published studies aimed at improving the activation and care of patients with diabetes in the United States, to our knowledge, no study has sought to enhance care of patients with diabetes using a fully comprehensive and adaptable MHCE-like system. We seek to demonstrate improvement in patient activation measured by the Patient Activation Measure (PAM) instrument [21,55]. We believe that, in improving their activation, patients will also realize an improvement in diabetes self-care activities measured by their Summary of Diabetes Self-Care Activities (SDSCA) [56] scores.

## Methods

### Trial Design

This is a multisite, phased study conducted within the MHS that includes a user-centered design phase and a PCMH-based feasibility trial. In phase 1, we will assess both patient and provider preferences regarding the enhancement of the MHCE technology capabilities for type 2 diabetes chronic care management. The phase 2 research will be a single-blinded (patients only) 12-month feasibility study that will incorporate randomization principles. We will employ a 1:1 allocation ratio between intervention and control.

### Inclusion and Exclusion Criteria

Inclusion criteria for patient participation in phase 1 or 2 research are the following: (1) men and women aged 18 years or older, (2) able to understand and read English, (3) enrolled for primary care to one of the target PCMH sites, and (4) having a diagnosis of type 2 diabetes. Additionally, with respect to phase 2 patients, we will seek to recruit a maximum of 120 (per site), with a distribution of patients with PAM levels 1-4, a sample representative of the patients enrolled in the PCMH. We did not derive the 120 per site recruitment numbers from power calculations, but deemed them to be sufficient. Finally, participants for phase 2 must be available for a 12-month study.

Inclusion criteria for clinician participation in phase 1 or 2 research are the following: (1) being a physician, physician assistant, nurse practitioner, or nurse employed at the target site, and (2) providing care for patients with type 2 diabetes.

Exclusion criteria for patient participation in phase 1 or 2 research are the following: (1) pregnant women, (2) non-English-speaking patients, (3) receiving hospice care, (4) having active cancer and receiving treatment with chemotherapy or radiation therapy, (5) taking warfarin, (6) recipient of gastric bypass or similar procedure, (7) having a diagnosis of uncontrolled hypothyroidism, (8) having known Cushing syndrome, (9) being treated with oral steroids, (10) having known liver disease, (11) having a current diagnosis of cognitive impairments that would interfere with use of technology, (12) having congestive heart failure, in New York Heart Association functional class 3 or 4, and (13) unable to use a mobile device due to cognitive or physical impairments during initial screening. We exclude pregnant women because they require careful monitoring due to potential medical complications for the woman and unborn child. While some mHealth studies seek to include additional exclusions based on age, educational level, or technical literacy, our research team rejected adding any additional exclusion criteria beyond the 13 listed above. We purposely seek the “average” patient with type 2 diabetes in the target population. Feedback from our clinician investigators and research staff at our clinical sites is encouraging that these patients will be capable of using the intervention technology.

Exclusion criteria for clinician participation in phase 1 or 2 research are the following: (1) not affiliated with the target site, and (2) not providing care for patients with type 2 diabetes.

### Participant Enrollment

We will recruit patients via review of the PCMH clinic schedule, referrals from providers, distributed posters and fliers, and population health databases. Potential participants will be prescreened through verification of the inclusion and exclusion criteria based on a medical record review. Interested participants will be scheduled for a screening visit with study staff to provide informed consent and be administered the PAM instrument. Patients’ PAM scores will place them in a stratified group, where they will be randomly allocated.

Clinicians practicing in the respective PCMH sites will be invited to participate by word of mouth from the site’s principal investigator; this is a convenience sample. The clinician participants who would like to participate in the study will meet with the senior research associate to review the minimal-risk information sheet to be included in the study. For phase 2, clinicians will sign an informed consent form. The clinician participants will not be blinded in the study, nor allocated to intervention or control groups.

### Setting and Site Selection

We seek to purposefully assess the MHCE implementation for diabetes care in 2 distinctly different PCMH environments and geographic locations. The risks of attracting very different populations are mitigated by rather comprehensive inclusion and exclusion criteria, which will ensure similarity regarding patient acuity. The patient base includes those on active duty, retirees, and dependents who have typically spent years in the military and have been stationed at various locations. Both of the selected facilities are federal facilities and operated by the MHS.

Madigan Army Medical Center, the US Army’s second largest military treatment facility located in Tacoma, Washington, is a tertiary facility with a level II trauma center and robust graduate medical education programs. They serve a patient base of approximately 118,000 patients; about 7500 (or >6%) are living with type 2 diabetes. Of the diabetes population, about 15% are active duty members or their dependents, and about 85% are retirees and their dependents. Over half of the patients with diabetes are 57-76 years of age. The study location within the medical center is a PCMH managed by the Department of Internal Medicine. There are approximately 14,300 patients enrolled in this PCMH supported by a staff of 77 (12 staff physicians; 8 residents) responsible for their care.

Mike O’Callaghan Federal Medical Center is a federal facility in the greater Las Vegas, Nevada area, that serves approximately 47,000 patients; about 4500 (or >9%) are living with type 2 diabetes. Of the diabetes population, about 4% are active duty members or their dependents, and about 96% are retirees and their dependents. Over 72% of the patients with diabetes are in their 60s or older. The study location within the medical center is a PCMH managed by the Department of Family Medicine. There are approximately 7500 patients enrolled in this PCMH supported by a staff of 62 (9 staff physicians; 26 residents) responsible for their care.

### Description of the Mobile Health Care Environment

**Figure 1:**
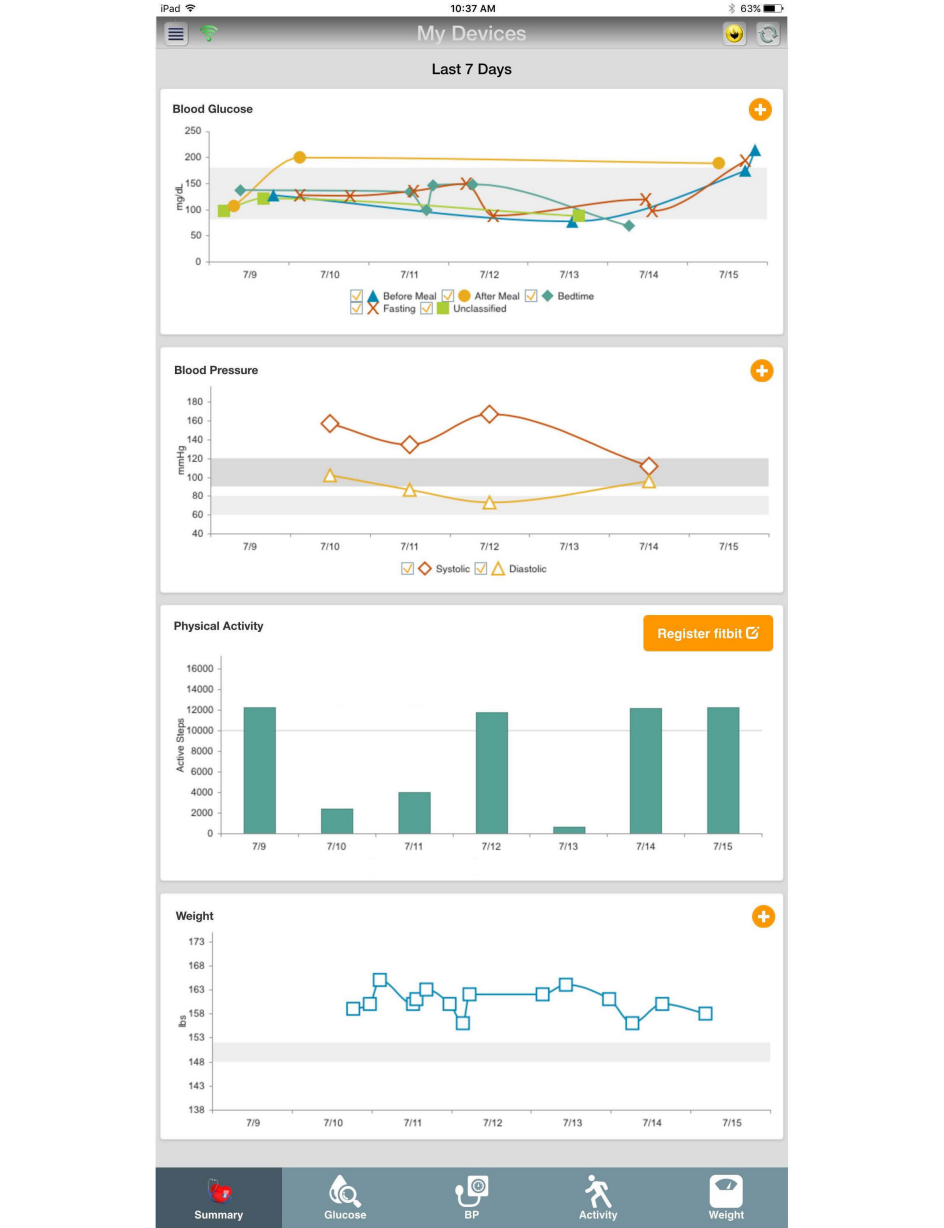
Mobile Health Care Environment home screen (patient view). BP: blood pressure.

The US Department of Defense (DoD) MHCE system is a secure health information system designed to support health services delivery and mHealth. The MHCE meets all physical and information security mandates, as prescribed by federal law and DoD regulation, for the protection of personal health information and personally identifiable information. The MHCE was developed by the DoD Telemedicine and Advanced Technology Research Center as a platform to support mHealth. Its first major application was to support patient engagement for wounded warriors rehabilitating in their communities. In the study, soldiers on average responded to ≥60% of weekly questionnaires related to behavioral health challenges, posttraumatic stress, or traumatic brain injury [57]. Our study is Telemedicine and Advanced Technology Research Center’s second major application. The MHCE is designed to remotely support patients by sending automated reminders, announcements, wellness tips, alerts, and status questionnaires. Figure 1 is a visual example of the graphical user interface that patients will see when accessing the MHCE. In this study, we enhance MHCE capabilities in several ways.

### Intervention Overview

Our intervention is based on an enhanced MHCE in several ways. First, we add the capability to include collection and visualization of data from Bluetooth-enabled medical devices. This includes mapping data from device output into the MHCE, developing data visualization appropriate for mHealth and clinical care (eg, graphing outcomes, temporal trend patterns), migrating data in an analysis cell, and developing decision-support algorithms that signal safety alerts and need for behavioral reinforcement. Devices used in this study include a scale, glucometer, blood pressure reader, and activity monitor. Second, we expand the capacity of the MHCE analysis cell to manage large amounts of data and to conduct both routine reports and research applications. Third, we add patient activation and associated measurement instruments for capturing baseline and ongoing changes to patient activation. Fourth, we expand the MHCE messaging platform that research associates, and later clinical support staff, can use to send tailored behavioral messaging to patients in an effort to influence greater activation and reinforce positive behavior.

The MHCE can be accessed by mobile phones or tablets that use either an IOS or Android platform. The MHCE requires Internet access for patients to sync data from devices (addressed above) to the MHCE backend portal, to receive tailored behavioral messages, or to use other functions. During the study, patients will additionally receive SMS messages with hyperlinks to a separate secure information system platform used for administration and analysis of PAM and SDSCA instruments. MHCE activity, or lack thereof, will be monitored by senior research associates, who can prompt patients via tailored behavioral messages or direct contact.

### Tailored Behavioral Messaging

A primary component of the MHCE system is tailored behavioral messaging. Tailored behavioral messages are more likely than generic messages to facilitate health behavior change when they are aligned with individuals’ beliefs, lifestyle, demographics, social norms, or interests [58-60].

In this study, the research team has developed behavioral messages tailored for each of the 4 PAM score levels; in total we have developed 360 messages. The messages fall within 9 functional areas common to diabetes care: nutrition, home monitoring, physical activity, blood pressure, foot care, medications, smoking, glucose control, and general behavioral reinforcement. The messages are consistent with general concepts and goals of self-management behaviors consistent with the DoD-Veterans Affairs clinical practice guideline for type 2 diabetes and the SDSCA survey instrument.

Since different PAM levels require different strategies, we addressed varying needs through a combination of applied constructs. Specifically, level 1 messages must address the emotional state of feeling overwhelmed and passive with an emphasis on the importance of taking action. To address the needs of PAM level 1 patients, we use constructs from social networks and social support theory [61], specifically that of emotional support that emphasizes expressions of empathy and caring. We encourage a sense of hope by expressing the belief and expectation that the message recipient can change his or her situation and overcome difficulties. Constructs from the transtheoretical model [62] such as visioning, dramatic relief, self-reevaluation, and environmental reevaluation also guided level 1 message development.

PAM level 2 messages build knowledge and self-efficacy to engage in a behavior and focus on ways to take small steps that don’t require much in-depth knowledge. Self-efficacy and the confidence a person feels about performing a particular activity was a primary construct used to develop these messages with a focus on one of the main strategies to build self-efficacy, that of taking small steps that are likely to result in performance accomplishment. Outcome expectations, or the anticipatory outcomes of a behavior, stated in ways that would likely appeal to the expectancies or values a person places on the outcome, was also an important construct [63].

PAM level 3 messages assume some knowledge and focus more on building self-management skills such as goal setting and self-monitoring. For messages in this level, we used transtheoretical model [62] constructs relevant to the preparation and action stages of behavior change.

PAM level 4 messages about staying the course and avoiding relapse when stressed were grounded in the transtheoretical model constructs guiding processes used in the maintenance stage of change. Also used in level 4 message development were strategies developed in a relapse prevention model [64] such as identifying high-risk situations for relapse and the development of specific coping strategies for those situations.

**Figure 2:**
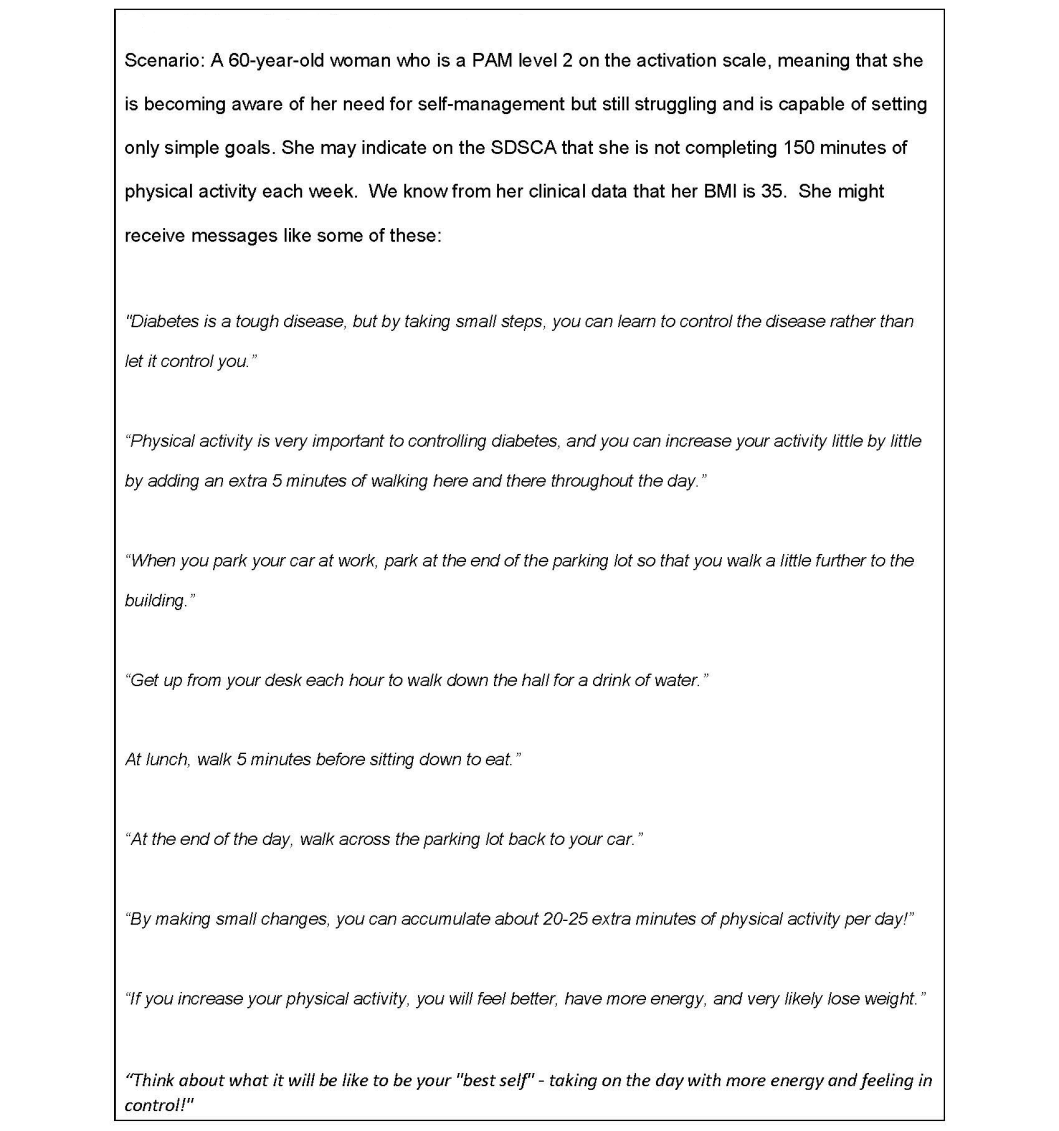
Example of tailored health messaging.

In phase 2 of our study, tailored behavioral messages will be sent to each intervention group participant, via the MHCE accessed through their mobile device, based on both senior research associate-initiated and algorithm-automated schedules and thresholds developed according to PAM level, SDSCA responses, and agreed-upon general rotation. Figure 2 offers examples. The senior research associates will use the MHCE backend portal control panel for manual rotational scheduling of messages to be delivered 3 days per week (typically Monday, Wednesday, and Friday) within the MHCE system. Participant responses to the SDSCA may trigger additional messaging if their clinical readings from biomedical devices exceed established safety thresholds.

### Phase 1 User-Centered Design Study Flow

In phase 1 we will evaluate and gain feedback from patients with diabetes regarding MHCE app navigation, use of external devices, ease of use, and satisfaction. We will collect baseline research participant data to include basic demographic data and clinical measures following verification of informed consent. One researcher-facilitator will lead individual participants through usability testing and the additional researcher-observer will document observations. During a facilitator-provided demonstration of the MHCE, the facilitator will ask each participant to concurrently navigate to each component of the MHCE system via a mini tablet device under their control. For each task, we will ask 3 open-ended questions to evaluate task-specific user satisfaction regarding the look and layout of the app, how the app functions, and any specific issues that are confusing. Next, the facilitator will give a brief demonstration of the external devices that will be used in the study: a blood pressure monitor, a glucometer, a digital precision weight scale, and a Fitbit Charge wireless activity and sleep wristband. For each device, we will ask participants to (1) manually upload data, (2) sync each device with the app, and (3) interpret graphs. While it would be preferable to observe the MHCE in the context of where the patient would actually use the system, financial limitations prohibit such expanded usability observation research.

Research staff will evaluate usability by applying definitions and usability evaluation metrics guided by the International Organization for Standardization’s 9241-11 usability framework and mHealth usability research [65]. Specific metrics to evaluate usability are effectiveness, efficiency, and satisfaction. We will evaluate effectiveness via task completion and error coding. We will assess timed task completion as a task being completed with ease, being completed with minor mistakes, or not completed. Errors will be coded using a codebook developed by the phase 1 team. The observer will also note when users commit errors they cannot solve or commit errors that prevent further progress. We will use the Single Ease Question to evaluate informant satisfaction immediately after performing each task [66]. The System Usability Scale (SUS) will evaluate overall informant satisfaction with the MHCE [67].

We will also assess provider preferences in phase 1 using focus groups of clinicians and nurses recruited from the 2 study sites. Two trained qualitative researchers will facilitate the focus groups. We will take field notes during the focus groups and audio record each session to ensure accuracy of the field notes. The facilitators will use a semistructured interview guide to elicit clinician and nurse feedback about the MHCE. After briefly demonstrating the app, facilitators will ask 6 broad questions (with probes), developed by the phase 1 team in conjunction with study coinvestigators. These questions are designed to elicit feedback from participants regarding app design, alerts (general), wording of alerts, perceived usefulness to patients for promoting self-management, clinical usefulness and workflow, and backend portal data summaries. We will probe specific issues related to clinical usefulness of the MHCE in the context of the clinical workflow of the PCMH environment.

A 4-member team will complete a thematically organized data analysis of the clinician and nurse feedback using an inductive narrative approach [68-70]. We will begin with an analysis of field notes from 1 randomly selected provider and 1 nurse to create an initial codebook. We will expand the codebook as we continue to code field notes. The analysis team will divide the coding duties so that each transcript is coded by 2 independent coders [71]. The team will meet during the coding process to address consensus, update the coding structure, and revisit any previously coded field notes that need to be reviewed again based on these updates. Codes will be applied to the transcripts using Atlas.ti software version 7.5.10. Codes drawn from the interview guide will serve as the organizing framework for analysis. As new themes emerge, we will expand the narrative.

### Phase 2 Controlled Study: Patient Enrollment and Study Flow

For phase 2 we will recruit 240 patients (120 per site), with half assigned as a control group. Eligible patients will be first assigned to 4 strata according to their PAM score. After all patients are identified and assigned into the strata, simple randomizations will be performed within each stratum to assign patients to either the MHCE or usual care groups. Patients will be randomly allocated to either the control or the intervention group based on their PAM scores.

We will modify the MHCE system between phase 1 and phase 2 research, incorporating phase 1 observations and optimizing system usability at the patient level. We will collect baseline research participant data, including basic demographic data and clinical measures, following verification of informed consent.

### MHCE Intervention Versus Usual Care

Patients in both the intervention and usual care (control) groups will receive a device package as outlined in Textbox 1. These devices will collect and record biometric data. All patients will be trained in using biomedical devices and peripheral equipment.

**Patient device package (intervention and control groups).**

- Activity monitor (Bluetooth and cloud enabled)
- Scale (Bluetooth enabled)
- Blood pressure cuff (Bluetooth enabled)
- Glucometer (Bluetooth enabled)

**Figure 3:**
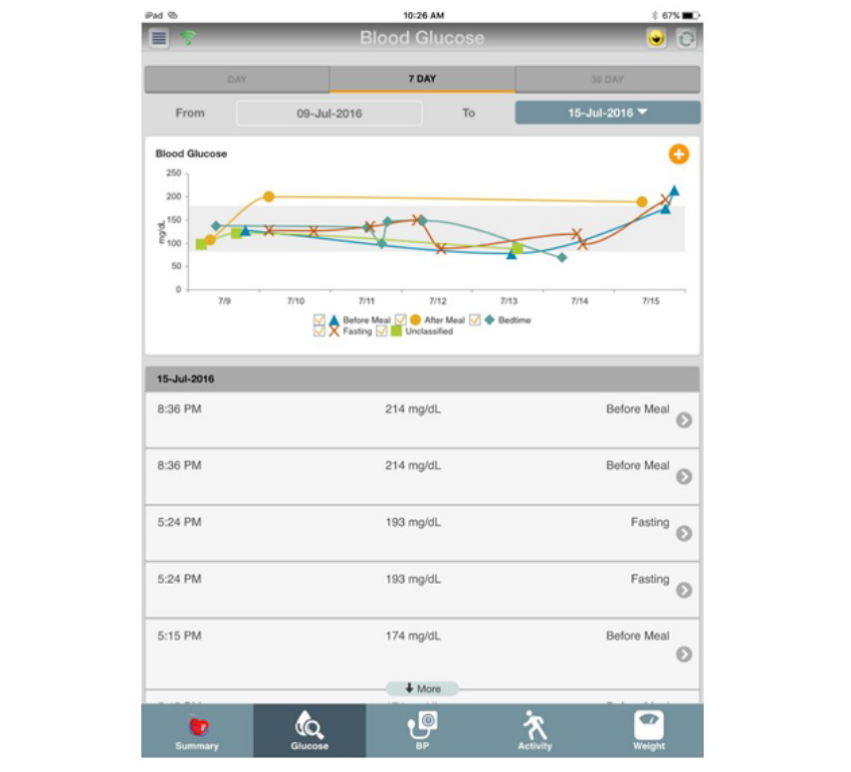
Example of visualization of patient data. BP: blood pressure.

For the patient groups allocated to the intervention, their devices will be mapped to the MHCE system accessible from the patients’ mobile phone or an iPad mini tablet device. Data from their biomedical devices will be visually presented in the MHCE with trend and scalable options (Figure 3).

Safety algorithms will be mapped to these clinical data to alert the patient and, depending on the measure, the clinical team when readings exceed established thresholds. The intervention groups will also have full access to and will receive the tailored behavioral messaging outlined above. At time of study enrollment, we will provide a tablet device to patients who are fully eligible to participate, are allocated to the intervention group, but do not have a mobile phone (with iOS or Android operating system).

In both the intervention and control groups, the patients’ clinician and PCMH support team will be notified of the patients’ enrollment in the study. The intervention patients will be encouraged to regularly use the MHCE system as a tool to improve their diabetes self-care.

### Initial Outcome Measures for Patient Component

Primary outcome measures are PAM scores. Secondary outcome measures in the study are (1) SDSCA responses, (2) clinical measures (Textbox 2), (3) comorbid conditions (eg, uncontrolled plasma glucose, hypertension, hyperlipidemia, stoke, eye disease, coronary heart disease), (4) SUS survey scores, (5) MHCE usage statistics, and (6) health services utilization measures.

**Clinical measures in phase 2.**

- Glycated hemoglobin (HbA_1c_)
- Low-density lipoprotein
- High-density lipoprotein
- Height and weight
- Abdominal circumference
- Systolic blood pressure
- Diastolic blood pressure

### Patient Activation Measure Instrument

**Figure 4:**
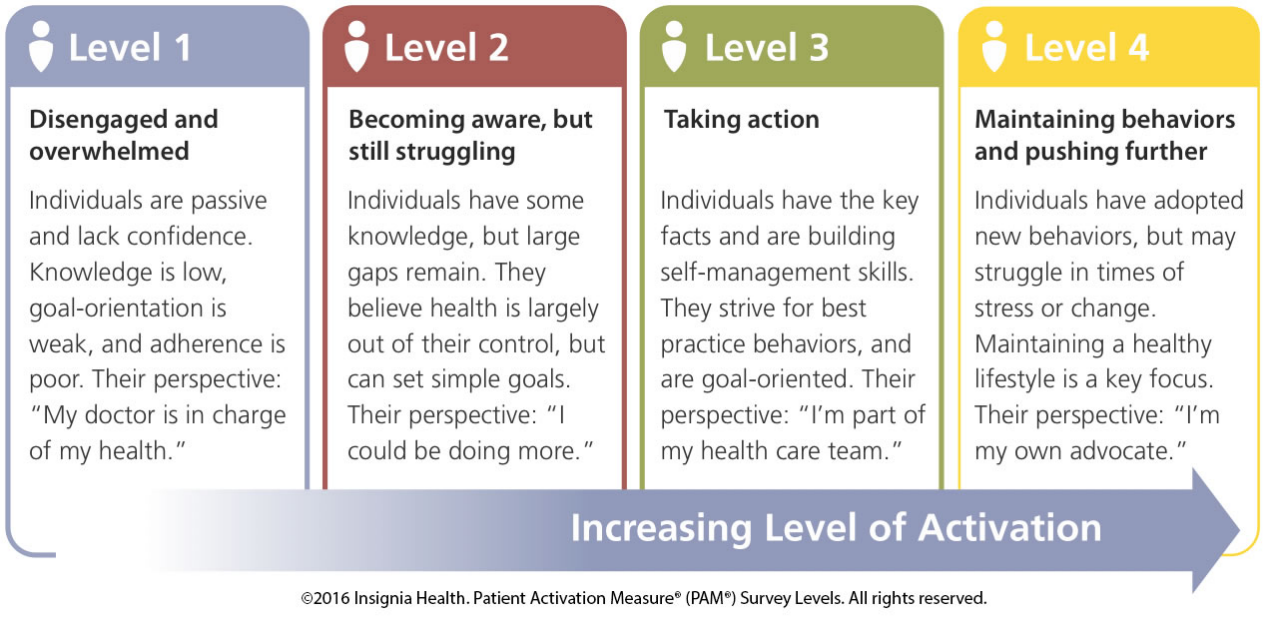
The 4 levels within the Patient Activation Measure (PAM) survey.

The self-reported PAM survey is associated with self-management behaviors, medication adherence, patient satisfaction, and quality of life [55,72]. Within a diabetes-specific population, PAM is not related to knowledge regarding HbA_1c_ (the standard measure of average blood glucose level [73]), but is associated with better glycemic control [74]. Interventions, including educational programs [31], care coaching [32], and motivational interviewing [33], have been attempted to improve this activation with varied success. Specifically, patient activation can be increased with targeted, patient-centered, repeated messaging [19]. The PAM is a valid, reliable, unidimensional, probabilistic Guttman-like scale that was validated over a decade ago [21] and is a standard tool to measure patient activation. We will administer the PAM at screening visits in phases 1 and 2, and electronically every 3 months during phase 2 for both the intervention and control groups. Figure 4 outlies the 4 PAM levels.

### Summary of Diabetes Self-Care Activities Instrument

The SDSCA instrument is a brief self-report instrument for measuring levels of self-management across different components of the diabetes regimen [56]. The SDSCA includes 11 core items associated with diabetes self-care. The SDSCA has been successfully used in numerous diabetes studies both within and outside the United States [56,75-78]. The SDSCA has been validated and is considered a standard instrument in diabetes care for measuring self-care activities, with its validation and reliability published nearly two decades ago [56]. We will administer the SDSCA at the intake visit for phase 2, and electronically every 2 weeks during phase 2 for both the intervention and control groups.

### Clinical Measures

We will collect clinical measures (Textbox 2) from patients at intake during phase 1 research. We will collect and compare changes in patient clinical measures for both groups in phase 2 at 3 points: intake, midpoint (month 6), and conclusion (month 12). For patients assigned to the MHCE intervention group, the MHCE system will also record weight, systolic blood pressure, diastolic blood pressure, and blood glucose values to the MHCE module on a regular basis via Wi-Fi or Bluetooth-enabled peripheral equipment.

### Clinician Support for Patient Activation Measure

The Clinician Support for Patient Activation Measure (CS-PAM) instrument measures clinician beliefs about patient self-management behavior. The CS-PAM has been a valid and reliable instrument in use since 2010 [25]. The CS-PAM score indicates an individual clinician’s overall level of endorsement or belief about the importance of patient self-management, as well as beliefs about the importance of specific patient competency categories [25].

In phase 2, we will measure clinician support for patient self-management by the CS-PAM. PCMH clinicians (ie, physicians, nurse practitioners, and physician assistants) in this study will take the CS-PAM at 3 points in the study: beginning, midpoint (month 6), and conclusion (month 12).

### System Usability Scale Survey

The SUS survey is a 10-item Likert-like scaled survey used to convey a subjective assessment of system usability. The instrument was developed over 15 years ago and is used to measure the usability of websites. The SUS was validated on several occasions, with perhaps the largest validation study (including 10 years’ worth of data) conducted in 2008 [79]. In this study we will substitute the term “MHCE system” for the term “website” in the instrument. We will conduct the SUS survey at the conclusion of the encounter for phase 1 patients, and at midpoint (months 5-6) and study conclusion (months 11-12) for phase 2 patients in the intervention group.

### MHCE Usage Statistics

Our technology enablement partners will embed counters (invisible to patients) that track usage of MHCE components. These counters will export usage data to our research analysis database. Summary statistics and trends will be analyzed with comparison.

### Comorbid Conditions

We will assess and document comorbid conditions (eg, hypertension, hyperlipidemia) among both the control and intervention groups during prescreening of eligibility, at intake, at study midpoint, and at study conclusion. While not primary outcome measures, any change over time and whether the number and type of comorbid conditions influence patient use of MHCE will be assessed.

### Data Analysis Strategy

We will conduct the primary analyses for phase 2 using an intent-to-treat approach. Study participants will be retained in their original assignment groups after the random allocation in the analysis. Achievement of randomization will be evaluated through the comparison of baseline key variables between the MHCE intervention group and the control group. We will also compare baseline key characteristics between eligible patients who participate in the study and those who do not participate to examine the potential for bias.

To test hypotheses 2 and 3, that patients who participate in MHCE will have higher PAM, SDSCA, and SUS scores and improved selected clinical outcomes and comorbid conditions than their counterparts in usual care, we will use multivariate regression models (logistic regression if the outcome is a binary variable and linear regression if the outcome is a continuous variable) with the intervention assignment as the primary independent variable. Stratified analyses will be conducted (eg, sex, race, and initial PAM score).

The primary comparison will be outcomes at 12 months. Additional analyses will use longitudinal analysis models using a generalized estimating equation, which will include outcomes at both 6 and 12 months.

To test hypothesis 4, that patients who engage at a higher rate with the interactive and tailored mobile technology in MHCE will realize greater improvement in clinical measures (eg, HbA_1c_ values; Textbox 2), we will use multivariate linear regression models. Clinical outcomes will be the dependent variables and will be tested separately. The main independent variable will be MHCE usage. We will examine the association between the dependent variable and MHCE usage by using the generalized estimate equation with adjustment of potential confounders (eg, age, sex, race, duration of disease, use or nonuse of insulin).

### Trial Status

At the time of publication, we have completed phase 1 data collection. Formal analysis of phase 1 data has not been completed. Institutional review board approval (study and site implementation) has been obtained and phase 1 research commenced in late fall 2016.

## Results

The hypotheses of the study suggest that patients can, and will, improve their activation in chronic care self-management. Improved activation should translate into improved diabetes self-care. While not powered in this study, improved self-management activities should lead to fewer emergency situations (and trips to the emergency department), weight loss (in many cases), improved blood pressure, and improved clinical measures. Cumulatively, the gains should translate into improved quality of life if our hypotheses are supported.

This study has been approved by the institutional review boards of Clemson University (protocol #IRB2015-234) and the Madigan Army Medical Center (representing both DoD sites; reference #216073). Study personnel will follow protocol with all informed consent mandates directed by the institutional review boards; informed consent in this study includes both patients and clinicians or key clinical staff. This trial was registered with ClinicalTrials.gov (NCT02949037) on October 31, 2016.

## Discussion

Expected benefits of this research and development effort to the scientific community and health care services include improved understanding of how to advance 3 joint PCMH principles (ie, better coordination of care, improved quality and safety, and enhanced access to care) through the use of mobile technology and improved understanding of how to include mHealth technology in the clinical workflow of the PCMH health services model, as well as improved understanding of how to use mHealth technology to activate patients with a diagnosis of type 2 diabetes in disease self-management behaviors. We also expect to improve understanding of how patient complexity and degree of “sickness” may influence patient use or nonuse of mHealth technologies in self-management of their disease, and to explore how to map patient-entered biomedical data onto clinical documentation and a decision-support platform useful in chronic care management.

Our study design is not immune from potential threats to validity. Patients allocated to the control arm will be issued the same peripheral devices as the intervention group and, while they may not achieve the same degree of activation, they may realize improvement if they use the equipment being issued to them. Though this behavioral mechanism could benefit patients in the control group, a strong activation change in the control arm could conceal the behavioral benefit of our intervention when we compare patient behavior from the 2 arms.

We are aware that we did not conduct a power calculation for sample size, since this project was funded as a feasibility study, not a randomized controlled trial. Thus, sample size estimates are neither required nor appropriate. We additionally recognize that a formal randomized controlled trial would be preferred to our current design. A follow-on randomized controlled trial is our goal once we have collected sufficient data and have a better understanding of how patients will use this chronic care health information technology system. At that point we will legitimately be able to predict the intervention effect and properly power the study.

## Abbreviations

CS-PAM: Clinician Support Patient Activation Measure
DoD: Department of Defense
HbA1c: glycated hemoglobin
MHCE: Mobile Health Care Environment
MHS: Military Health System
PAM: Patient Activation Measure
PCMH: patient-centered medical home
PHR: personal health record
SDSCA: Summary of Diabetes Self-Care Activities
SMS: short message service
SUS: System Usability Scale
